# Do black women’s lives matter? A study of the hidden impact of the barriers to access maternal healthcare for migrant women in South Africa

**DOI:** 10.3389/fsoc.2024.983148

**Published:** 2024-05-30

**Authors:** Abha Jaiswal, Lorena Núñez Carrasco, Jairo Arrow

**Affiliations:** ^1^Bronx Community College (BCC), City College of New York (CUNY), New York, NY, United States; ^2^University of the Witwatersrand, Johannesburg, South Africa; ^3^Independent Researcher, Montevideo, Uruguay

**Keywords:** migrant women, maternal mortality, South Africa, xenophobia, African migrant

## Abstract

**Background:**

Studies on the barriers migrant women face when trying to access healthcare services in South Africa have emphasized economic factors, fear of deportation, lack of documentation, language barriers, xenophobia, and discrimination in society and in healthcare institutions as factors explaining migrants’ reluctance to seek healthcare. Our study aims to visualize some of the outcome effects of these barriers by analyzing data on maternal death and comparing the local population and black African migrant women from the South African Development Countries (SADC) living in South Africa. The heightened maternal mortality of black migrant women in South Africa can be associated with the hidden costs of barriers migrants face, including xenophobic attitudes experienced at public healthcare institutions.

**Methods:**

Our analysis is based on data on reported causes of death (COD) from the South African Department of Home Affairs (DHA). Statistics South Africa (Stats SA) processed the data further and coded the cause of death (COD) according to the WHO classification of disease, ICD10. The dataset is available on the StatsSA website (http://nesstar.statssa.gov.za:8282/webview/) for research and statistical purposes. The entire dataset consists of over 10 million records and about 50 variables of registered deaths that occurred in the country between 1997 and 2018. For our analysis, we have used data from 2002 to 2015, the years for which information on citizenship is reliably included on the death certificate. Corresponding benchmark data, in which nationality is recorded, exists only for a 10% sample from the population and housing census of 2011. Mid-year population estimates (MYPE) also exist but are not disaggregated by nationality. For this reason, certain estimates of death proportions by nationality will be relative and will not correspond to crude death rates.

**Results:**

The total number of female deaths recorded from the years 2002 to 2015 in the country was 3740.761. Of these, 99.09% (*n* = 3,707,003) were deaths of South Africans and 0.91% (*n* = 33,758) were deaths of SADC women citizens. For maternal mortality, we considered the total number of deaths recorded for women between the ages of 15 and 49 years of age and were 1,530,495 deaths. Of these, deaths due to pregnancy-related causes contributed to approximately 1% of deaths. South African women contributed to 17,228 maternal deaths and SADC women to 467 maternal deaths during the period under study. The odds ratio for this comparison was 2.02. In other words, our findings show the odds of a black migrant woman from a SADC country dying of a maternal death were more than twice that of a South African woman. This result is statistically significant as this odds ratio, 2.02, falls within the 95% confidence interval (1.82–2.22).

**Conclusion:**

The study is the first to examine and compare maternal death among two groups of women, women from SADC countries and South Africa, based on Stats SA data available for the years 2002–2015. This analysis allows for a better understanding of the differential impact that social determinants of health have on mortality among black migrant women in South Africa and considers access to healthcare as a determinant of health. As we examined maternal death, we inferred that the heightened mortality among black migrant women in South Africa was associated with various determinants of health, such as xenophobic attitudes of healthcare workers toward foreigners during the study period. The negative attitudes of healthcare workers toward migrants have been reported in the literature and the media. Yet, until now, its long-term impact on the health of the foreign population has not been gaged. While a direct association between the heightened death of migrant populations and xenophobia cannot be established in this study, we hope to offer evidence that supports the need to focus on the heightened vulnerability of black migrant women in South Africa. As we argued here, the heightened maternal mortality among migrant women can be considered hidden barriers in which health inequality and the pervasive effects of xenophobia perpetuate the health disparity of SADC migrants in South Africa.

## Introduction

The impact of racism, discrimination, and xenophobia on the health of minority and migrant groups health has been addressed in the literature (Shaeffer, 2009; Groos et al., 2018; Andaya, 2019; Braveman and Parker Dominguez, 2021). The effects of structural racism and discrimination against specific groups often result in the worsening of health conditions; it affects chronic and infectious diseases (Groos et al., 2018) and impacts mental health (Patrão et al., 2019) and maternal health (Jewkes et al., 1998; Kruger and Schoombee, 2010; Adams 2014; Valdez and Deomampo, 2019; Tenorio et al., 2022). Researching migrants’ health to identify the impact of racism and xenophobia on migrants’ morbidity is a complex task. It needs to consider the epidemiological profile of the population in the host society, the relationship between the host society and migrant groups, and other factors that shape health in particular ways and relate to the mobility of the migrant population (Abubakar et al., 2018).

In terms of the epidemiological profile of South Africa, the HIV/AIDS epidemic has greatly impacted death rates in the adult population in the country over the last three decades. In 2000, AIDS accounted for 25% of all deaths, and AIDS mortality was 3.5 times higher than in 1985 among 25–29-year-old women and two times higher among 30–39-year-old men (Dorrington et al., 2001). AIDS deaths increased exponentially over the following 5 years to reach a peak in 2006 (with 282,904 AIDS deaths).^1^ In July 2002, when the Constitutional Court judgment ordered the government to make nevirapine universally available, ARVs started to become available in clinics all over South Africa (Benatar, 2004). In 2021, 7.5 million people are living with HIV and nearly 75% are on ART coverage.^2^ The country has the largest ARV program in the world, its constitution ensures everyone living in South Africa should be able to access ARV treatment (Department of Health South Africa, 2007). While accessing treatment has significantly decreased AIDS deaths, including maternal deaths, the distribution of these deaths among the population reveals the hidden side of health inequality (National Department of Health, 2011; South African National AIDS Council, 2012). Overall, women have a higher biological vulnerability to HIV/AIDS infection. In a previous study (Carrasco et al., 2020), it was found that the proportion of women dying of infectious diseases in South Africa was higher than that of men, irrespective of nationality. Yet, black migrant women encounter additional barriers to accessing ARV treatment in the country. Studies have shown that treatment is often denied to migrants on account of documentation, and the nurses directly refer migrants to the NGO sector to access treatment (Vearey, 2008; Vearey, 2023). Many barriers, including racism and xenophobia, are preventing black women migrants from accessing healthcare, and this affects their health in general and increases preventable deaths among this population. Against this backdrop, we explore the possibility of barriers to healthcare by examining the disparity in maternal deaths among SADC migrants living in South Africa.

The differential impact of barriers to access to reproductive healthcare for black women has become apparent in various contexts (Andaya, 2019; Valdez and Deomampo, 2019). A study conducted in Brazil shows an increase in maternal mortality among black women during the pandemic of COVID-19. As the authors explain, “the experience of becoming pregnant and giving birth has been a risky practice for adolescents, women, and black people who are pregnant. They are discriminated against, humiliated, misguided, and do not receive quality information to live this stage of life with dignity” (Tenorio et al., 2022). A comprehensive report on maternal and child health in South Africa conducted in 164 health centers in 2008 highlighted five main causes of maternal deaths that help elucidate what can be the specific impact on migrant women. These include non-pregnancy-related infections such as AIDS, tuberculosis, and pneumonia contributed at 38%, followed by hypertension and hemorrhage at 19 and 13%, respectively, 8% of maternal deaths were attributed to sepsis, and 6% to pre-existing medical diseases (South Africa Every Death Counts Writing Group, 2008). While the situation indicates the need to strengthen the healthcare system, key gaps in access were attributed to (1) administrative weaknesses such as poor transport facilities; (2) lack of healthcare facilities and appropriately trained staff; (3) patient-oriented problems such as no antenatal care (ANC) or infrequent ANC attendance and delay in seeking medical help; (4) health worker-oriented problems such as healthcare provider failed to follow protocol (delay in referring patients) and poor initial assessment and recognition/diagnosis; and (5) communication problem (Mabaso et al., 2014). It can be inferred that the first two attributes of the healthcare system would have a bearing on every person accessing the healthcare system, while the latter three causes would have a greater impact on healthcare access among migrant women and translate into a lower ANC attendance. Studies have confirmed that there is an association between high maternal mortality and no attendance to ANC in the country, resulting in “the women not having attended the appropriate level of care during pregnancy and childbirth, and not having received interventions during pregnancy such antiretroviral treatment” (Gumede et al., 2017).

Maternal mortality is considered a key indicator of access to adequate and quality health services. Differences in the number of maternal deaths among various groups are a demonstration of social inequities where racial and class divides are key categories to understand the difference in access to healthcare (Coovadia et al, 2009). It has been shown that distance from health facilities affects access, which relates to class and race. McLaren et al. (2014) demonstrate how respondents in the poorest income quintiles live 0.5–0.75 km further from the nearest health facility. Furthermore, racial differentials in the likelihood of having a health consultation or an attended birth persist even after controlling for confounders (McLaren et al., 2014). Preventable maternal, neonatal, and child (MNC) deaths are unacceptably high in South Africa (South Africa Every Death Counts Writing Group, 2008). These deaths, most of which are preventable, have increased over the last two decades (Statistics South Africa, 2022). The pregnancy-related mortality ratio reported in the South African District Health Survey (SADHS) in 2016 was 536 deaths per 100,000 live births. This represents a statistically significant increase from 1998 when the pregnancy-related mortality ratio reported in the SADHS was 150 deaths per 100,000 live births (2016 South African District Health Survey).

An unexplored dimension of health inequality is the impact of the divide in accessing healthcare on migrants’ morbidity. We argue that the ultimate impact of this disparity is reflected in avoidable deaths affecting black foreign women in South Africa, and this hidden reality needs to be made visible. While numerous studies highlight the barriers to healthcare that migrants face, studies that quantify the long-term impact of the barriers to access healthcare on health outcomes are non-existent (Rees et al., 2017). Our study aims to estimate the effect of the barriers to accessing healthcare among women migrants by analyzing statistics on maternal deaths among migrant women from SADC countries. As we compare these deaths with South African women’s deaths, we want to make visible the burden of xenophobic violence on black migrant women. The question we address in this study is the extent to which being a black migrant woman in South Africa increases the risk of maternal mortality.

Historically, migrants form an integral part of South Africa. The established democracy, economic stability, and good infrastructure lure migrants into the country (Adepojou, 2006). Since the end of Apartheid, the number of migrants in the country has doubled. In 2019, migrants in South Africa were estimated at 4.2 million and women represented 43.1% of the migrant population (Global Migration Data Portal, 2020). For migrants, the complex pattern of socioeconomic exclusion generated by migrant status adversely affects them at multiple levels. There are only a few studies that investigate in detail infectious diseases and the sexual and reproductive health of migrants. Of these, one was conducted in Hillbrow (Gumede et al., 2017; Rees et al., 2017), a migrant-dense area of inner Johannesburg. The authors showed that since their arrival, migrant women have been alienated, and many have had to turn to sex work for a living due to a lack of other “alternatives” (Gumede et al., 2017; Rees et al., 2017). Moreover, it has been seen that regardless of women migrants residing in the Hillbrow being young and better educated than their local counterparts, their contact with healthcare providers was limited for fear of deportation due to their legal status, eviction from their homes, stigma, and xenophobia (Rees et al., 2017). The pervasiveness of these barriers to access will be further discussed in detail.

### Barriers to access healthcare for black migrant women

It has been established that primary healthcare clinics frequented by migrants had both the lowest levels of antenatal care (ANC) attendance and low levels of HIV testing and the highest level of HIV prevalence compared to clinics attended by both migrants and South African nationals (Gumede et al., 2017). The lack of ANC attendance among migrants results in high risk, further affecting birth outcomes adversely. Studies have shown that the odds of stillbirths and poor health outcomes are higher among women attending a lesser number of ANC visits (McDiehl et al., 2021). Although the availability of insurance increases the likelihood of attending ANC visits (Sakeah et al., 2017), the absence of such a safety net perpetuates the vulnerability and marginalization of migrants and adds to the barriers to accessing care.

There is rampant political scapegoating in South Africa in which foreign nationals, particularly those from Africa, are seen as flooding the country, taking people’s jobs, and overwhelming the public healthcare system (Mawadza and Crush, 2010; Crush and Tawodzera, 2014). Often expressed in the popular discourse, South African nationals believe foreigners ‘use up’ state resources and that they bring diseases when they come to South Africa (Crush and Tawodzera, 2014; Vearey et al., 2017). Many South Africans believe that migrants, especially refugees and the undocumented, should not receive healthcare in South Africa (Landau 2006; Bloch, 2010).

The media has a role in spreading false information that migrants are too many, that they are traveling to South Africa for health-seeking purposes, overburdening the South African public health system, and, in this way, they have shown to be instrumental in framing discourses that fuel xenophobia in South Africa (Danso and McDonald, 2001; Cruch, 2008; Mawadza and Crush, 2010). Labeled as “aliens,” “illegals,” and “foreigners,” migrants are perceived as “bringers of disease and crime, takers of jobs or consumers of ‘our’ resources” (Mawadza and Crush, 2010, p. 363). These terms permeate public discourse, fuelling xenophobia and anti-foreigner sentiments. It has been observed that xenophobia has become institutionalized in South Africa, a country that has been described as one of the most xenophobic in the world (Crush and Tawodzera, 2014). Scholars argue that the unchecked dislike and hostility for foreigners in South Africa contributed to the 2008 violence and, later, in 2015, fueled the everyday acts of violence that migrants experience in the country. While attention has been placed on the bodies of migrants affected by the violence, not much has been said about the violence on the bodies of women and their children in healthcare institutions. Not only do xenophobic sentiments manifest in the streets, but they are also carried to workplaces by government officials who encounter foreign nationals seeking public services, including healthcare institutions (Moyo, 2010; Crush and Tawodzera, 2014). Because the victims are often poor black Africans, some scholars and opinion leaders have argued that more than xenophobia, there is Afrophobia. Studies that focus on the experiences of migrant women accessing sexual and reproductive health services in South Africa have reported various challenges, including language barriers, maltreatment, neglect, demand for documents, fear of arrest, and outright denial of services (Lefko-Everett, 2007; Munyewende et al., 2011; Makandwa and Vearey, 2017; Chekero and Ross, 2018).

### Fear of arrest and deportation

A study conducted in the town of Makhado, in Limpopo province, shows that women migrants were very wary of visiting public healthcare facilities due to fear of being arrested in route to the hospital/clinic or being turned in by healthcare workers if they discover they do not possess proper documentation that legitimizes their stay in the country (Lefko-Everett, 2007). The same was observed in research conducted in Giyani, also in the Limpopo province, where women migrants reported that when overstayed the days allocated to them at the border by immigration officials, they tried as much as possible to reduce contact with the public officials for fear of being arrested and deported back to Zimbabwe (Chekero and Ross, 2018). Others (Crush and Tawodzera, 2014) also observe that visits to medical facilities or a mere venture into the city provide a platform for confrontation with the policing authorities. In Chekero and Ross’s study, the migrants’ accounts were corroborated by frequent law enforcement presence at the city’s entrance, the main aim of which was to look for migrants without proper documents. Thus, silence, secrecy, and invisibility were the norms for the women migrants, and this proved to be a health penalty since policing of the streets translated into policing of the healthcare facilities—policing of the streets negotiated and moderated their access to healthcare facilities.

Elsewhere, Lefko-Everett gives an account of a young migrant woman who, because of fear of arrest and deportation, tragically lost her baby as these factors prevented her from seeking maternal healthcare services (Lefko-Everett, 2007). Fears for arrest and deportation were common among female migrant sex workers in Cape Town and Johannesburg who approached healthcare facilities with caution for fear of being arrested and deported to their countries of origin (Richter et al., 2014). Such fears mean women migrants have two layers of inhibiting factors, i.e., the police in the streets and healthcare workers who sometimes act as immigration officials by asking for identity documents. These fears also cut across the general migrant populace whose irregular immigration status stands as a barrier between them and much-needed healthcare.

### Demand for documentation

In an earlier study, Moyo (2010) shows how frontline healthcare workers demand documents from foreign patients as a precondition to access healthcare in an inner-city clinic. Later, Chekero and Ross’s study focuses on the request for “papers” (documentation) at healthcare institutions and the implications of not having them in navigating health-help-seeking in the public sector. They observe that “migrants quickly learnt that possessing ‘right papers’ (resident permits, refugee status or asylum-seeking permit) is critical in accessing the state health sector” (Chekero and Ross, 2018). Crush and Tawodzera report that treatment for Zimbabweans was denied upon failing to produce proper documents—including proof of residence, even in cases of emergencies or severe illnesses (Crush and Tawodzera, 2014). Denial of treatment on account of documentation is not only limited to minor illnesses but extends to chronic illnesses such as HIV. Vearey observed that antiretroviral treatment (ART) was denied to migrants on account of documentation, and the nurses directly referred migrants to the non-governmental organization (NGO) sector to access treatment (Vearey, 2008; Vearey et al., 2011). Fuller (2008) also reports on migrant women’s challenges, which include being turned away from facilities due to a lack of proper documentation.

### Language

Language has been made a barrier as migrants fail to understand the service providers (Lefko-Everett, 2007; Vearey, 2023). Language is used as a mechanism to discern the nationality of patients; if migrants fail to speak the South African native languages and use English, they have reported being subjected to verbal abuse from nurses (Makandwa and Vearey, 2017). Language cannot be separated from neglect, inattention, and maltreatment that the migrant patient experiences when accessing public health services in South Africa. Researchers have observed that once it is discovered that patients are Zimbabwean nationals, all courtesy to the patient is shelved (Crush and Tawodzera, 2014). The use of the English language, as opposed to a native language, exposes the nationality. Failure to communicate in local languages leads to ridicule, and nurses make it a point that they do not communicate in English even if they are capable of doing so. Language is used as an identifier of one’s nationality to set the platform for the orchestration of segregation and alienation (Crush and Tawodzera, 2014).

### Triaging of patients based on nationality

Apart from language, migrant women and migrants, in general, reported that South African citizens are given preference at the facilities (Lefko-Everett, 2007). Edwards (2009) speaks of triaging—a principle of prioritizing patient treatment based on the severity and kind of their illness. Crush and Tawodzera (2014) use this concept to explain the preferential treatment given to South African patients over migrants. They argue that the triaging of patients at health institutions is based not on health status but on race, origin, and language. Foreignness results in neglect, while citizenship engenders preferential treatment.

All of this sets the tone and platform for a better understanding of other challenges that black migrant women face when they approach public healthcare facilities for healthcare services. Other challenges that can be understood using this lens are what others have referred to as insensitivity and social exclusion (Munyewende et al., 2011). Munyewende et al. (2011, p. 5) also chronicle challenges such as “confusion about eligibility for treatment and negative, unfriendly attitudes from facility staff.” Rumors of unpleasant previous experiences (Scorgie et al., 2011; Vearey, 2012) are said to travel fast among the migrant community, which leads to migrant women developing a strong suspicion of the South African public healthcare system (Crush and Tawodzera, 2014; Chekero and Ross, 2018; Vearey et al., 2018).

By focusing on maternal mortality in South Africa, our study aims to visualize some of the most dramatic effects of these barriers. Based on existent Stats SA data on causes of death, our analysis compares the trends in maternal mortality among South African citizens (RSA) and African migrants from countries that are part of the South African Development Community (SADC) for the years 2002–2015. Migrants from these countries constitute nearly 70% of the migrants in the country.

## Materials and methods

Our analysis is based on data on reported causes of death (COD) from the South African Department of Home Affairs (DHA). Statistics South Africa (Stats SA) processed the data further and coded the cause of death (COD) according to the WHO classification of disease, ICD10. The dataset is available on the StatsSA website^3^ for statistical purposes only. The entire dataset consists of over 10 million records and about 50 variables of registered deaths that occurred in the country between 1997 and 2018. In our analysis, we have used data from the years 2002–2015, the years for which information on citizenship is reliably included on the death certificate. Corresponding benchmark data, in which nationality is recorded, exists only for a 10% sample from the population and housing census of 2011. Mid-year population estimates (MYPE) also exist but are not disaggregated by nationality. For this reason, certain estimates of death proportions by nationality will be relative and will not correspond to crude death rates.

We have selected the population group of migrants from the SADC countries for our study, as they constitute most migrants in South Africa, representing 68% of the total international migrants in South Africa (Stats SA, Census 2011). Within this group, we have included Zimbabwe, Swaziland, Botswana, Lesotho, Mozambique, Namibia, Mauritius, Angola, Zambia, and Malawi. Tanzania and Madagascar were excluded from our study as the data for those deaths was shown to be inconsistent during the study period. In addition, we also excluded data where the sex codes were missing.

In what now follows, we will summarize and describe data according to several variables of interest to our study. Table 1 gives the number of DHA recorded deaths alongside the estimated deaths and total population from the corresponding Stats SA mid-year estimate for the same year.

**Table 1:** Number of estimated Stats SA MYPE deaths, DHA recorded deaths (COD), and Stats SA MYPE total population: 2002–2015.

Year	Stats SA MYPE deaths	DHA-reported deaths (COD)	Stats SA MYPE—total population
2002	581,147	516,700	45,920,831
2003	619,789	573,139	46,460,581
2004	648,774	594,299	47,020,713
2005	661,940	613,506	47,601,909
2006	671,812	628,632	48,204,889
2007	660,794	620,595	48,830,411
2008	634,042	613,239	49,479,270
2009	602,288	598,291	50,152,301
2010	574,718	566,712	50,850,383
2011	551,597	531,840	51,574,437
2012	550,702	509,994	52,325,433
2013	535,958	492,261	53,104,386
2014	538,866	492,048	53,912,366
2015	532,761	487,607	54,750,491

**GRAPH 1:**
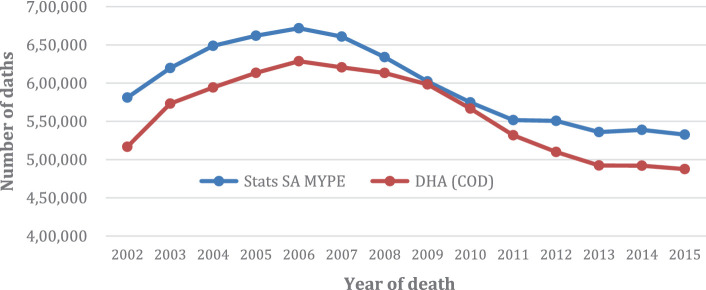
Number of Deaths from Stats SA MYPE and DHA COD: 2002–2015.

In Graph 1, shown below, we have plotted the corresponding number of deaths, both reported and estimated, covering the study period 2002–2015. MYPE and DHA reported deaths show similar trends and therefore was used as a benchmark to test for data integrity.

Under Stats SA, causes of death data (COD) is coded by Stats SA following the International Classification of Diseases, tenth revision (ICD-10) used by the World Health Organization (2016). We considered the *underlying cause of death* for our analysis, which is defined as “the disease or injury which initiated the train of morbid events leading to death or circumstances of the accidents or violence which produced the fatal injury” (22). Under the ICD 10 codes, we selected the codes assigned to pregnancy-related causes of maternal mortality (O00-O99). We have analyzed pregnancy-related deaths for women of reproductive ages (between the ages of 15 and 49 years) for three reasons. First, pregnancy is the period when women usually avail healthcare services. Second, maternal mortality is a key indicator of access to adequate and quality healthcare services. Third, studies by Mabaso et al. (2014) have highlighted factors that reveal deficiencies in the healthcare system, namely administrative weaknesses such as poor transport facilities, lack of healthcare facilities, and appropriately trained staff affecting all patients. In addition, there are other factors stressed by these authors that, although not exclusively, are likely to affect foreign migrants’ patients more adversely; these include patient-oriented problems such as no antenatal care (ANC) or infrequent ANC attendance and delay in seeking medical help; health worker-oriented problems such as healthcare provider failed to follow protocol leading to delay in referring patients and poor initial assessment and recognition/diagnosis; and communication problems. We argue that the attitudes of healthcare workers toward foreign patients could act as a proxy for barriers to access to healthcare affecting migrant women, a phenomenon that, as discussed earlier, has been termed as medical xenophobia. Focusing our study on pregnancy-related deaths can assist in quantifying the systemic barriers that migrant women face when availing of pregnancy-related care.

Except for the information from the 2011 census, there is a lack of information on the size of the total migrant population and the various national communities, limiting the possibility of calculating the death rate. To address this limitation, we used the percentage of death attributable to maternal mortality of the total female deaths to compare among SADC women migrants and South African women, revealing the variability in patterns of death along with temporal trends among these two groups. The difference in the crude deaths appears statistically significant. We further wanted to test if there was a significant difference in the odds of dying from pregnancy-related causes between individuals of different nationalities using logistic regression. We set up a binary logistic regression model with the outcome variable being the binary indicator of whether the cause of death is related to pregnancy or not. The predictor variable of interest is the binary indicator of nationality (SADC = 1 and RSA = 0 being the reference group).

Table 2 shows the deaths for the period 2002–2015 according to the cause of death and nationality.

**Table 2:** Female deaths by citizenship and cause of death (15–49 years).

Citizenship	South African	SADC	Total
Pregnancy-related	17,228 (97.4%)	467 (2.6%)	17,695
Not pregnancy related	1,513,267 (98.7%)	20,295 (1.3%)	1,533,562
Total	1,530,495 (98.7%)	20,762 (1.3%)	1,551,257

The variable Y is the binary outcome variable (1 = death from pregnancy-related disease, 0 = death from other causes). Meanwhile, X is the binary predictor variable indicating nationality (1 for a female from SADC and 0 for a South African female in the age group 15–49 years).

We, therefore, used logistic regression to model the probability of death from maternal causes according to the nationality of the deceased. The event (death due to pregnancy-related causes) being modeled is the underlying cause of death so that the outcome variable Y is binary and takes a value of 1 if the cause is death due to pregnancy-related causes and 0 otherwise. Similarly, for nationality, the independent variable X, we assign a value of 1 if the deceased is a SADC migrant and 0 if the deceased is South African. Finally, the odd ratio was used to test the statistical significance of the results.

## Results

### Overall female deaths for the period between 2002 and 2015

The total number of all female deaths recorded from the years 2002 to 2015 in the country was 3740.761. Of these, 99.09% (*n* = 3,707,003) were deaths of South African women and 0.91% (*n* = 33,758) were deaths of SADC female citizens (Table 3).

**Table 3:** No of female deaths and percentages for all causes of death for all age groups by nationality (2002–2015).

Year of death	SADC	South African	Total
2002	2,386 (0.96)	245,072 (99.04)	247,458
2003	2,334 (0.84)	274,208 (99.16)	276,542
2004	1,931 (0.67)	288,064 (99.33)	289,995
2005	2,227 (0.74)	298,507 (99.26)	300,734
2006	1.787 (0.59)	302,846 (99.41)	304,633
2007	2,158 (0.72)	297,409 (99.28)	299,567
2008	2,642 (0.89)	293,506 (99.11)	296,148
2009	2,734 (0.95)	284,479 (99.05)	287,213
2010	2,624 (0.97)	268,902 (99.03)	271,526
2011	2,612 (1.04)	247,827 (98.96)	250,439
2012	2,740 (1.16)	232,880 (98.84)	235,620
2013	2,457 (1.08)	225,864 (98.92)	228,321
2014	2,644 (1.10)	224,589 (98.84)	227,233
2015	2,482 (1.10)	222850 (98.90)	225,332
Total	SADC	South African	Total

**GRAPH 2:**
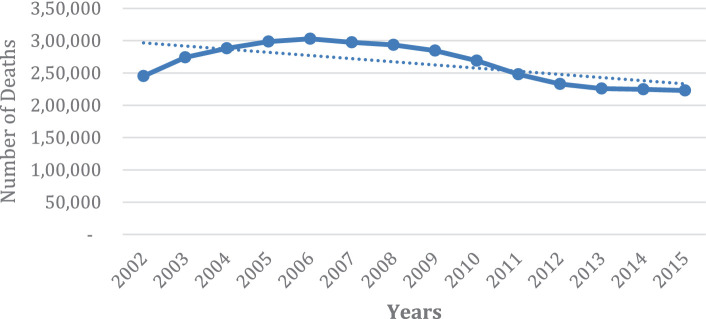
Number of deaths of women, all ages, RSA for all causes of death (2002–2015).

**GRAPH 3:**
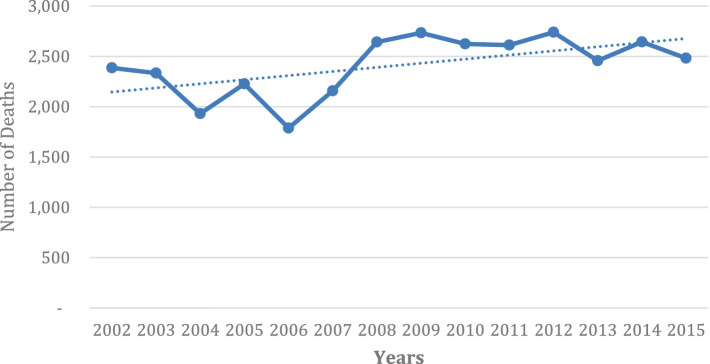
Number of deaths of SADC women, all ages, for all causes of death (2002–2015).

As observed in Graphs 2, 3, among South African women (RSA), the period 2002–2006 shows a rising trend in death, after which there is a sharp decline in mortality. The number of deaths stabilizes from 2013 onwards. Thereafter, the overall mortality trend continues to decline but less sharply. The sharp decline after 2006 could be attributed to the universal rollout of ARVs in the public healthcare system in the country from 2002 onwards. However, in contrast, among SADC women, mortality decreased slightly from 2002 to 2004, after which the mortality of this population group seems to show an upward trend. More importantly, the trends are consistently higher. Indeed, the number of deaths registered in 2015 is comparable to the number of deaths registered in 2004. The number of deaths ranged from 2000 to 2,500. Graphs 2, 3 of all causes of mortality for RSA and SADC migrants highlight the contrasting trends in female mortality between the two groups for the period of the study.

### Overall maternal mortality trends in South Africa for women aged 14–59 years (2002–2015)

A total of *n* = 1,530,495 deaths were recorded for women between the ages of 15–49 years of age. Of these, deaths due to pregnancy-related causes contributed approximately 1% of these deaths. South Africa contributed to *n* = 17,228 deaths, and SADC maternal deaths were *n* = 467 during the period under study (Table 4).

**Table 4:** Maternal mortality due to pregnancy-related causes among women, 15–49 years, from RSA and SADC countries (2002–2015).

	Pregnancy-related deaths		All female deaths aged 15–49		Percentage of pregnancy-related deaths to all deaths aged 15–49	
Death year	RSA	SADC	RSA	SADC	RSA pregnant %	SADC pregnant %
2002	789	12	109,629	1,174	0.71	1.02
2003	883	13	127,052	1,234	0.69	1.05
2004	1,152	10	138,598	1,045	0.83	0.95
2005	1,226	23	141,397	1,229	0.86	1.87
2006	1,354	23	140,210	1,260	0.96	1.82
2007	1,722	28	133,605	1,553	1.28	1.80
2008	1,728	52	128,319	1,863	1.34	2.79
2009	1,830	45	119,025	1,706	1.53	2.63
2010	1,593	52	108,075	1,724	1.47	3.01
2011	1,172	39	91,842	1,583	1.27	2.46
2012	975	38	81,915	1,671	1.19	2.27
2013	918	37	74,826	1,494	1.22	2.47
2014	985	47	69,685	1,648	1.41	2.85
2015	901	48	66,317	1,578	1.35	3.04
Total	17,228	467	1,530,495	20,762		

**GRAPH 4:**
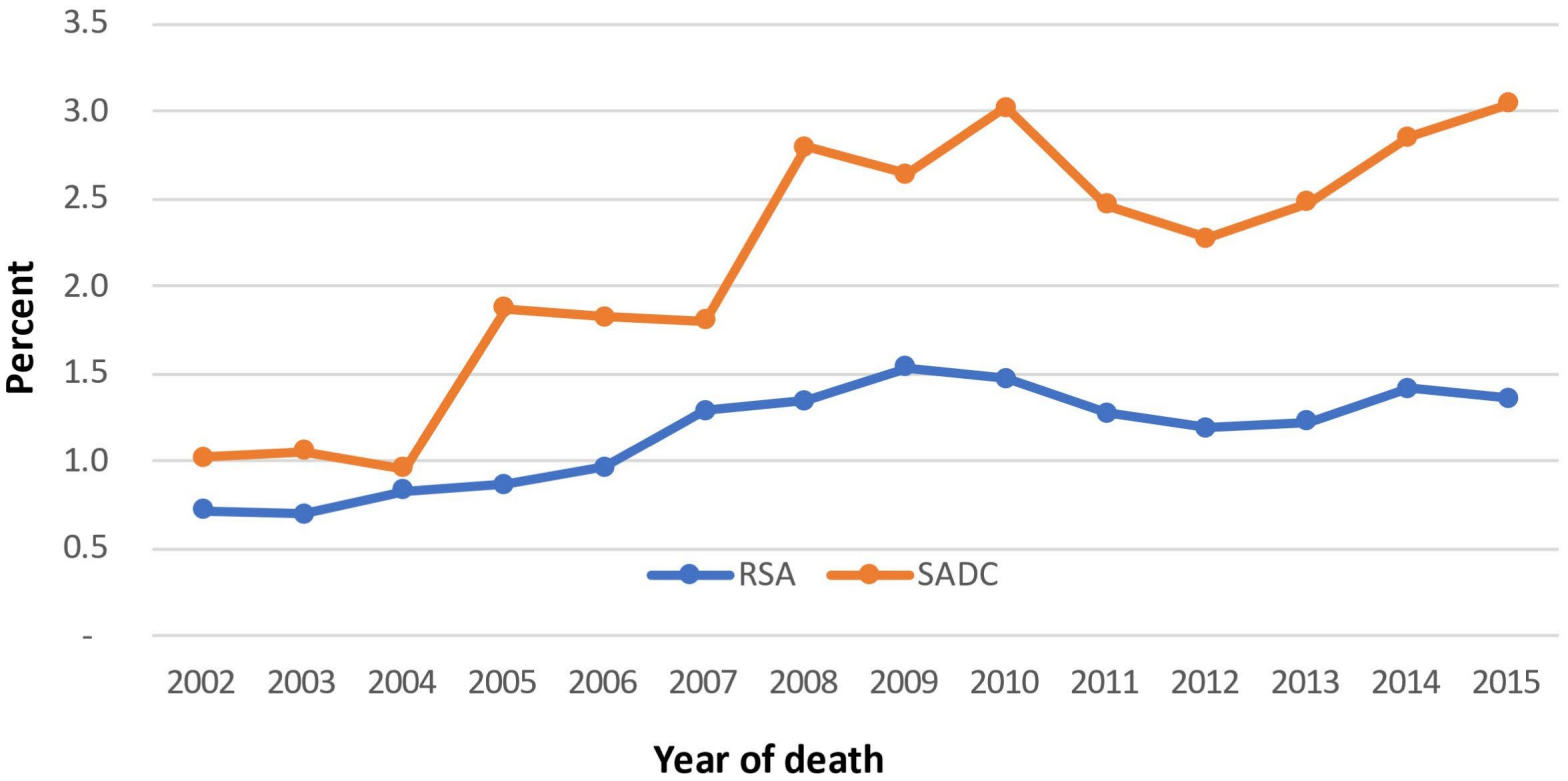
Percentage of pregnancy-related deaths, women aged 15–49 years by nationality (2002–2015).

As shown in Graph 4, the percentage of pregnancy-related maternal deaths among women aged 15–49 years is consistently higher for women from SADC countries than for South African women during the study period. The only significant decline in these deaths for women from SADC countries is in the years 2003 to 2004. From 2005 to 2008, an uptick in the percentage of maternal deaths was observed, followed by a decline in the percentage of maternal deaths. The trend reversed from 2012, and these deaths continued to increase till 2015. The decline in the percentage of maternal deaths is not consistent for South African women; there is no substantial decrease in maternal death among SADC women except for the years 2011–2013. In terms of the trends, the disparity in percentages of maternal deaths among these two groups increased during the study period. In 2015, the percentage of deaths among SADC migrant women was nearly 50% higher than among South African women.

The logistic regression was used to estimate the odds of dying from a pregnancy-related cause when comparing SADC women to South Africans. The logistic regression analysis showed that the probability of a migrant female dying from a pregnancy-related cause was greater than that for a South African. The odds ratio was 2.02. In other words, the odds of a migrant female dying were more than twice that of a South African. This result is statistically significant as this odds ratio, 2.02, falls within the 95% confidence interval (1.82–2.22).

## Summary of findings

Our analysis departed from examining all female deaths for the years 2002–2015 and compared these for SADC women and women from RSA. The findings show contrasting trends in female mortality between these two groups for the study period. Death among SA women has decreased over the period in the study; in contrast, among SADC women, mortality seems to show an upward trend. Indeed, the number of deaths in 2015 is comparable to the number of deaths in 2004. Regarding pregnancy-related deaths, our analysis of the age group aged between 15 and 49 years old showed that this cause of death is 50% higher among women from SADC countries than among women from South Africa. There is no certainty about the reason for the heightened mortality among SADC migrants in 2015. However, as background information, it should be noted in 2015 that xenophobic attacks erupted in Durban and spread to major cities such as Johannesburg. Under these circumstances, fear of accessing healthcare could have led to increased preventable deaths. Again, using logistic regression, the odds of a migrant female from SADC countries to find death due to maternal-related causes were more than twice that of a South African. This result is statistically significant as this odds ratio, 2.02, falls within the 95% confidence interval (1.82–2.22).

## Discussion

Studies on the barriers migrant women face when trying to access healthcare services in South Africa have emphasized economic factors, fear of deportation, lack of documentation, language barriers, and importantly xenophobic attitudes from healthcare providers. These factors are referred to as explaining migrant women’s reluctance to seek healthcare. Yet, a proper assessment that considers the effect of these barriers on the worsening of health conditions and migrants’ mortality has not been conducted. This study offers insights into the possible implications of the barriers to accessing healthcare in the lives of black migrant women. Through this comparison of the causes of death of black migrant women from SADC countries with South African women, we aim to visualize the possible far-reaching adverse effects of barriers to access healthcare and xenophobia on the health and wellbeing of black migrant women and their families. This study has shed light on what could be the ultimate impact of societal relationships and institutional discrimination against black migrants and how health inequality is reproduced, impacting black women’s lives and that of their children in the Southern African Region. This study contributes to the growing number of students across regions that aim to visualize the health impact of racism, discrimination, and xenophobia on minority and migrant groups globally.

The fear of availing of family planning services and sexual and reproductive health services not only affects women’s health but also has a bearing on the health of the newborn. Studies have shown that unplanned pregnancies are often associated with reduced odds of breastfeeding and increased odds of the infant’s health being compromised (Kost and Lindberg, 2015). Stats SA has made an important contribution to the study of morbidity in the country by offering data on death disaggregated by nationality, which is analyzed in this study. It is imperative that, as per the provisions spelled out in the South African constitution, measures should be taken to ensure migrants feel safe to use the healthcare system that demonstrates the lives of black women and their children matter.

## Conclusion

The study is the first to examine and compare data on maternal death of women from SADC countries and of South African women and was done based on Stats SA data available for the years 2002–2015. This analysis allows for a better understanding of the differential impact that social determinants of health have on mortality among black migrant women in South Africa and considers access to healthcare as a determinant of health. As we examined maternal death, and while the data does not allow for the establishment of causal relations, it could be inferred that the heightened mortality among black migrant women in South Africa was associated with barriers to access to healthcare, such as xenophobic attitudes of healthcare workers toward foreigners during the study period of the study. Although the negative attitudes of healthcare workers toward migrants have been reported in the literature and the media, until now, its long-term impact on the health of the foreign population has not been measured. While a direct association between heightened death and xenophobia cannot be established in this study, we hope to offer evidence that supports the need to focus on the heightened vulnerability of black migrant women in South Africa. As we argued here, the heightened mortality among migrant women should be considered hidden forms of xenophobia and barriers to healthcare services, which have perpetuated health disparities for migrants from SADC counties in South Africa. As future research, we would like to stress the importance of conducting qualitative studies that help us understand the causes of premature death and heightened mortality of migrants. It is equally important to have access to reliable data. The data provided by STATS SA on the causes of death categorized by nationality is of great significance. However, to determine mortality rates, it is essential to have information on the number of migrants in the country. Regular monitoring of such data is crucial to assess the level of risk that migrants in South Africa face concerning premature death.

## Data availability statement

Publicly available datasets were analyzed in this study. This data can be found here: Stats SA Causes of death 2002–2015, http://nesstar.statssa.gov.za:8282/webview/.

## Author contributions

AJ: Writing – original draft. LN: Writing – review & editing. JA: Writing – review & editing.

## Footnotes

^1^ https://www.statista.com/statistics/1331607/number-of-deaths-from-aids-in-south-africa/#:~:text=In%202022%2C%20the%20estimated%20number,in%20South%20Africa%20reached%2085%2C796

^2^ https://kpatlas.unaids.org/dashboard

^3^ http://nesstar.statssa.gov.za:8282/webview/

## References

[ref1] Abubakar I. Aldridge R. W. Devakumar D. Orcutt M. Burns R. Barreto M. L.. (2018). The UCL–Lancet Commission on migration and health: the health of a world on the move. Lancet 392, 2606–2654. doi: 10.1016/S0140-6736(18)32114-7, PMID: 30528486 PMC7612863

[ref2] Adams J. H. (2014). Factors influencing migrant maternal and infant nutrition in Cape Town, South Africa. 198.

[ref3] Adepojou A. (2006). Internal and international migration within Africa. In: Migration in south and southern Africa: Dynamics and determinants, edited by Kok P. Oucho J. Gelderblom D. Zyl J. van. Cape Town: Human Sciences Research Council, 26–46.

[ref4] Andaya E. (2019). Race-ing time: clinical temporalities and inequality in public prenatal care. Med. Anthropol. 38, 651–663. doi: 10.1080/01459740.2019.1590826, PMID: 30950643

[ref5] Benatar S. R. (2004). Health care reform and the crisis of HIV and AIDS in South Africa. N. Engl. J. Med. 351, 81–92. doi: 10.1056/NEJMhpr033471, PMID: 15229313

[ref6] Bloch A. (2010). The right to rights? Undocumented migrants from Zimbabwe living in South Africa. Sociology 44, 233–250. doi: 10.1177/0038038509357209

[ref7] Braveman P. Parker Dominguez T. (2021). Abandon “race.” focus on racism. Front. Public Health 9:689462. doi: 10.3389/fpubh.2021.6894625 34557466 PMC8452910

[ref8] Carrasco L. N. Jaiswal A. Arrow J. Muteba M. K. Aryal B. (2020). Do migrants' lives matter? A comparison of causes of death between African migrants from SADC countries and south Africans in the post-apartheid era (2002-2015). Int. J. Migrat. Health Soc. Care 16, 459–468. doi: 10.1108/IJMHSC-08-2020-0078

[ref9001] Census (2011). Population dynamics in South Africa P Lehohla - Statistics South Africa, 2015 - statssa.gov.za.

[ref9] Chekero T. Ross F. C. (2018). “On paper” and “having papers”: Zimbabwean migrant women’s experiences in accessing healthcare in Giyani, Limpopo province, South Africa. Anthropol. Southern Africa 41, 41–54. doi: 10.1080/23323256.2018.1442729

[ref10] Coovadia H. Jewkes R. Barron P. Sanders D. McIntyre D. (2009). The health and health system of South Africa: historical roots of current public health challenges. Lancet 374, 817–834. doi: 10.1016/S0140-6736(09)60951-X, PMID: 19709728

[ref9002] Crush J. (2008). The Perfect Storm: The Realities of Xenophobia in Contemporary South Africa, Southern African Migration Programme (SAMP). Canada Retrieved from: https://policycommons.net/artifacts/1448552/the-perfect-storm/2080328/

[ref11] Crush J. Tawodzera G. (2014). Medical xenophobia and Zimbabwean migrant access to public health Services in South Africa. J. Ethn. Migr. Stud. 40, 655–670. doi: 10.1080/1369183X.2013.830504

[ref12] Danso R. McDonald D. A. (2001). Writing xenophobia: immigration and the print Media in Post-Apartheid South Africa, vol. 48: Indiana University Press, 24.

[ref13] Department of Health South Africa (2007). HIV and AIDS and STI strategic plan for South Africa, 2007–2011. Pretoria: Department of Health.

[ref14] Dorrington R. Bourne D. Bradshaw D. Laubscher R. Timæus I. M. (2001). The impact of HIV/AIDS on adult mortality in South Africa. Cape Town: Medical Research Council, 1–56.

[ref15] Edwards M. (2009). Hospital and home rehabilitation did not differ in functional competence in activities of daily living. Evid. Based Nurs. 12:84. doi: 10.1136/ebn.12.3.84 19553421

[ref16] Fuller R. (2008) Double jeopardy: women migrants and refugees in South Africa. Perspectives 3: 7–11.

[ref9003] Global Migration Data Portal (2020). Migration data portal. Available at: https://www.migrationdataportal.org/

[ref17] Groos M. Wallace M. Hardeman R. Theall K. (2018). Measuring inequity: a systematic review of methods used to quantify structural racism. J. Health Disparit. Res. Pract. 11:Article 13,

[ref18] Gumede S. Black V. Naidoo N. Chersich M. F. (2017). Attendance at antenatal clinics in inner-city Johannesburg, South Africa and its associations with birth outcomes: analysis of data from birth registers at three facilities. BMC Public Health 17:443. doi: 10.1186/s12889-017-4347-z, PMID: 28832284 PMC5498856

[ref19] Jewkes R. Abrahams N. Mvo Z. (1998). Why do nurses abuse patients? Reflections from south African obstetric services. Soc. Sci. Med. 47, 1781–1795. doi: 10.1016/S0277-9536(98)00240-8, PMID: 9877348

[ref20] Kost K. Lindberg L. (2015). Pregnancy intentions, maternal behaviors, and infant health: investigating relationships with new measures and propensity score analysis. Demography 52, 83–111. doi: 10.1007/s13524-014-0359-9, PMID: 25573169 PMC4734627

[ref21] Kruger L. Schoombee C. (2010). The other side of caring: abuse in a south African maternity ward. J. Reprod. Infant Psychol. 28, 84–101. doi: 10.1080/02646830903294979

[ref22] Landau L. B. (2006). Protection and dignity in Johannesburg: shortcomings of South Africa’s urban refugee policy. J. Refug. Stud. 19, 308–327. doi: 10.1093/jrs/fel012

[ref23] Lefko-Everett K. (2007). Voices from the margin: migrant women’s experiences in Southern Africa. Southern African Migration Programme (SAMP). Canada. Retrieved from: https://policycommons.net/artifacts/1448582/voices-from-the-margins/2080359/

[ref24] Mabaso M. Ndaba T. Mkhize-Kwitshana Z. (2014). Overview of maternal, neonatal and child deaths in South Africa: challenges, opportunities, Progress and future prospects Int. J. MCH AIDS. Available at: https://www.ncbi.nlm.nih.gov/pmc/articles/PMC4948143/ (Accessed April 22, 2022). PMC4948143 27621971

[ref25] Makandwa T. Vearey J. (2017). Giving birth in a foreign land: exploring the maternal healthcare experiences of Zimbabwean migrant women living in Johannesburg, South Africa. Urban Forum 28, 75–90. doi: 10.1007/s12132-017-9304-5

[ref26] Mawadza A. Crush J. (2010). Metaphors of migration: Zimbabwean migrants in the south African media. Available at: https://idl-bncidrc.dspacedirect.org/bitstream/handle/10625/44852/IDL-44852.pdf.

[ref27] McDiehl B. Mugyenyi G. Siedner M. Riley L. Ngonzi J. Bebell L. (2021). Antenatal care visit attendance frequency and birth outcomes in rural Uganda: a prospective cohort study. Matern. Child Health J. 25, 311–320. doi: 10.1007/s10995-020-03023-0 33201450 PMC7878332

[ref9004] McLaren Z. M. Ardington C. Leibbrandt M. (2014). Distance decay and persistent health care disparities in South Africa BMC Health Serv Res. 14:541. doi: 10.1186/s12913-014-0541-1 25367330 PMC4236491

[ref28] Moyo K. (2010). Street-level interface: the interaction between health personnel and migrant patients at an inner-city public health facility in Johannesburg. MA thesis in forced migration and displacement studies. University of the Witwatersrand.

[ref29] Munyewende P. Rispel L. C. Harris B. Chersich M. (2011). Exploring perceptions of HIV risk and health service access among Zimbabwean migrant women in Johannesburg: a gap in health policy in South Africa? J. Public Health Policy 32, S152–S161. doi: 10.1057/jphp.2011.36, PMID: 21730988

[ref30] National Department of Health (2011). Health data advisory and coordination committee report. Pretoria: Department of Health.

[ref31] Patrão A. L. Almeida M. Matos S. Goes E. F. (2019). Association between perceived discrimination and alcohol and tobacco consumption in ELSA-Brasil cohort: focusing on gender differences. Subst. Use Misuse 54, 1214–1225. doi: 10.1080/10826084.2019.1573838, PMID: 30799670

[ref32] Rees H. Delany-Moretlwe S. Scorgie F. Luchters S. Chersich M. (2017). At the heart of the problem: health in Johannesburg's inner-city. BMC Public Health 17:554. doi: 10.1186/s12889-017-4344-2, PMID: 28832289 PMC5498860

[ref33] Richter M. Chersich M. F. Vearey J. Sartorius B. Temmerman M. Luchters S. (2014). Migration status, work conditions and health utilization of female sex Workers in Three South African Cities. J. Immigr. Minor. Health 16, 7–17. doi: 10.1007/s10903-012-9758-4, PMID: 23238581 PMC3895178

[ref34] Sakeah O. Oduro A. Shibanuma A. Ansah E. Kikuchi K. Gyapong M. O.-A.. (2017). Determinants of attending antenatal care at least four times in rural Ghana: analysis of a cross-sectional survey. Glob. Health Action 10:1291879. doi: 10.1080/16549716.2017.1291879, PMID: 28578634 PMC5496066

[ref35] Scorgie F. Nakato D. Akoth D. O. Netshivhambe M. Chakuvinga P. Nkomo P.. (2011). “I expect to be abused and I have fear”: Sex workers’ experiences of human rights violations and barriers to accessing healthcare in four African countries. 77.

[ref36] Shaeffer R. (2009). No Healing Here [electronic Resource]: Violence, Discrimination and Barriers to Health for Migrants in South Africa. USA: Human Rights Watch.

[ref37] South Africa Every Death Counts Writing Group. (2008). Every death counts use mortality audit data for decision-making to save the lives of mothers, babies, and children in South Africa. South Africa: Lancet. Available at: https://pubmed.ncbi.nlm.nih.gov/18406864/10.1016/S0140-6736(08)60564-4 18406864

[ref38] South African National AIDS Council (2012). National strategic plan on HIV, STIs and TB, 2012–2016 South Africa: South African National AIDS Council.

[ref39] Statistics South Africa. (2022) Africa SS. Available at: https: www.statssa.gov.za/?cat=19.

[ref40] Tenorio D. De Matos Brasil A. Nogueira B. Rolim Neto M. (2022). High maternal mortality rates in Brazil: inequalities and the struggle for justice. Lancet Reg. Health Am. 14:100343. doi: 10.1016/j.lana.2022.100343, PMID: 35935590 PMC9346430

[ref42] Valdez N. Deomampo D. (2019). Centring race and racism in reproduction. Med. Anthropol. 38, 551–559. doi: 10.1080/01459740.2019.1643855, PMID: 31829735

[ref43] Vearey J. (2008). Migration, access to ART, and survivalist livelihood strategies in Johannesburg. Afr. J. AIDS Res. 7, 361–374. doi: 10.2989/AJAR.2008.7.3.13.660, PMID: 25875464

[ref44] Vearey J. (2012). Learning from HIV: exploring migration and health in South Africa. Glob. Public Health 7, 58–70. doi: 10.1080/17441692.2010.549494, PMID: 21360380

[ref45] Vearey J. (2023). Migration and health in the WHO-AFRO region: a scoping review. South Africa: African Centre for Migration & society (ACMS), WITS University and World Health Organization Regional Office for Africa.

[ref46] Vearey J. de Gruchy T. Kamndaya M. Walls H. L. Chetty-Makkan C. M. Hanefeld J. (2018). Exploring the migration profiles of primary healthcare users in South Africa. J. Immigr. Minor. Health 20, 91–100. doi: 10.1007/s10903-016-0535-7, PMID: 27909937 PMC5772125

[ref47] Vearey J. Modisenyane M. Hunter-Adams J. (2017). Towards a migration-aware health system in South Africa: A strategic opportunity to address health inequity. 10.

[ref48] Vearey J. Richter M. Núñez L. Moyo K. (2011). South African HIV/AIDS programming overlooks migration, urban livelihoods, and informal workplaces. Afr. J. AIDS Res. 10, 381–391. doi: 10.2989/16085906.2011.637741, PMID: 25865514

[ref9005] World Health Organization (WHO) (2016). The ICD-10 classification of mental and behavioural disorders. Statistical Classification of Diseases and Related Health Problems 10th Revision, Available at: https://icd.who.int/browse10/2016/en.

